# The FANMI (“my FAMILY” in Creole) study to evaluate community-based cohort care for adolescent and young women living with HIV in Haiti: protocol for a randomized controlled trial

**DOI:** 10.1186/s12889-019-8065-6

**Published:** 2019-12-30

**Authors:** Grace Seo, Joseph Marie Bajo Joseph, Nancy Confident, Esther Jean, Bianca Louis, Tatiana Bell, Rose Cardelle Riche, Marie Elmase Belizaire, Vanessa Rouzier, Alexandra Apollon, Lindsey Reif, Vanessa Rivera, Elaine Abrams, Heejung Bang, Bruce Schackman, Daniel Fitzgerald, Jean W. Pape, Margaret L. McNairy

**Affiliations:** 1 Center for Global Health, Weill Cornell Medical College, New York, NY USA; 2 Haitian Group for the Study of Kaposi’s Sarcoma and Opportunistic Infections (GHESKIO), Port-au-Prince, Haiti; 3 Department of Epidemiology, Mailman School of Public Health, Columbia University, New York, NY USA; 4 Division of Biostatistics, Department of Public Health Sciences, University of California, Davis, CA USA; 5 Healthcare Policy and Research, Weill Cornell Medical College, New York, NY USA; 6 Department of Medicine, Weill Cornell Medical College, New York, NY USA

## Abstract

**Background:**

Adolescent girls and young women living with HIV in resource-limited settings have the poorest health outcomes of any age group, due in part to poor retention in care. Differentiated models of HIV care that target the specific challenges of young people living with HIV are urgently needed.

**Methods:**

The FANMI study is an unblinded randomized controlled trial designed to evaluate the efficacy of an adolescent-specific model of HIV care in Port-au-Prince, Haiti. The FANMI intervention places newly young women living with HIV who are not currently on ART or on ART ≤ 3 months, in cohorts of 5–10 peers to receive monthly group HIV care in a community location. In contrast, participants in the standard care arm receive routine HIV care and individual counseling each month in GHESKIO’s Adolescent Clinic. A total of 160 participants ages 16–23 years old are being randomized on a 1:1 basis. The primary outcome is retention in HIV care defined as being alive and in care at 12 months after enrollment. Secondary outcomes include viral suppression at 12 months, sexual risk behaviors, acceptability of the FANMI intervention, and health care utilization and costs.

**Discussion:**

The FANMI study evaluates a novel community-based cohort model of HIV care aimed at improving retention in care and reducing risk behaviors for HIV transmission among adolescent girls and young women living with HIV. Specifically, the FANMI model of care addresses social isolation by placing participants in cohorts of 5–10 peers to provide intensified peer support and makes HIV health management a group norm; reduces stigma and improves convenience by providing care in a community setting; and integrates clinical care and social support by the same providers to streamline care and promote long-term patient-provider relationships. If shown to be effective, the FANMI intervention may serve as a model of HIV care for improving retention among hard-to-reach adolescents and young adults in Haiti and could be adapted for other high-risk groups globally.

**Trial registration:**

Identifier: NCT03286504, Registered September 18, 2017.

## Background

Adolescents and youth account for over 30% of all new HIV infections globally [1]. It is estimated that 590,000 adolescents and youth between the ages of 15 to 24 were newly infected with HIV in 2017, and that up to 58% are females [2–4].. If linked to and retained in care, adolescents and youth living with HIV (AYLWH) have a near normal life expectancy [3]. However, multiple studies conducted in resource-limited settings report poor retention among this population, which contributes to an increase in morbidity and mortality [5–9]. Over the past decade, AIDS-related deaths among AYLWH has decreased by only 18% compared to a 48% decrease among adults [2].

Barriers to retention in care in many resource-limited settings include stigma, social isolation, and lack of family and peer support, as well as clinic-related factors such as disjointed care, long wait times, and lack of longitudinal relationships with providers. Moreover, the period of adolescence and youth are marked by significant physical, psychological, and social changes that influence one’s decision-making skills, risk perception, sexual behavior, and retention in care [10]. Adolescent girls and young women face additional challenges including gender violence, gender inequality, lack of access to education, transactional and age-disparate sex, and limited autonomy [11]. Novel approaches to HIV care are urgently needed to address the confluence of individual, clinic-related, developmental, and gender-related challenges that adolescent girls and young women face after an HIV diagnosis.

Haiti has the highest burden of HIV/AIDS in the Caribbean, with approximately 150,000 people living with HIV in 2017 [12]. Over 40% of new HIV infections in Haiti occur among adolescents and youth, 80% of which occur among young women [13]. GHESKIO (French acronym for the Haitian Group for the Study of Kaposi’s Sarcoma and Opportunistic Infections) is the largest HIV care provider in the Caribbean and is located in downtown Port-au-Prince. In 2010, GHESKIO implemented a youth-friendly Adolescent Clinic with the goal of providing services that specifically address the needs of AYLWH. After opening this clinic, rates of linkage to HIV care and initiation of antiretroviral therapy (ART) increased, but long-term retention in care remained poor [13]. In response, the FANMI model of HIV care was developed with the goal of improving retention in care [14].

We describe the design of the FANMI trial, which evaluates a novel and pragmatic model of HIV care for adolescent girls and young women in Haiti.

### Development of FANMI intervention

**Fig. 1:**
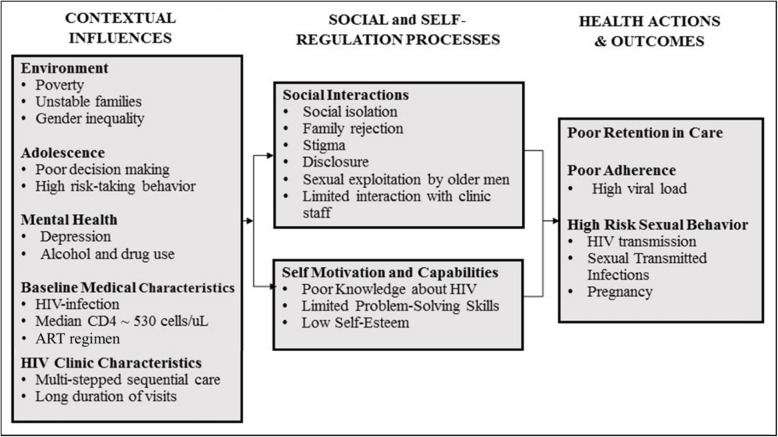
Adapted Social Action Theory: Factors Influencing Retention of Adolescent Girls and young Women Living with HIV in Haiti

The FANMI model of care was designed based on individual interviews and focus groups with patients, providers, and GHESKIO’s adolescent community advisory board (CAB), which identified key barriers to adolescent retention in care. Figure 1 illustrates the contextual, social, and individual factors influencing retention among adolescents and youth living with HIV using the adapted social action theory. This theory emphasizes the context in which behavior occurs and the confluence of social interaction and self-regulatory processes that affects health outcomes [15]. Young girls living with HIV reported profound social isolation, family rejection, and stigma associated with attending a specialized HIV clinic. They also felt “dread” coming to the medical clinic as it reminded them of their disease. During clinic visits, they reported that their visits consisted of a series of short encounters with multiple health care providers – i.e. various combinations of a nurse, a clinician, a counselor, a phlebotomist, and a pharmacist. The FANMI model of care was developed to address the following barriers: 1) social isolation and lack of family support; 2) stigma associated with clinic-based care; and 3) disjointed care from multiple providers. The adolescent CAB named this model of care “FANMI,” which is Haitian Creole for “FAMILY.”

## Methods

### Study design and objectives

FANMI is an unblinded randomized controlled trial of 160 females 16–23 years old who are randomized to the FANMI model (intervention) or to standard care (control). The study objective is to evaluate the efficacy of the FANMI model compared to standard care on the primary outcome of retention in HIV care at 12 months after enrollment. Secondary outcomes include HIV-1 RNA viral load suppression, sexual risk behaviors, FANMI acceptability among participants and providers, and health care utilization and costs (Table 1).

**Table 1:** Spirit flow diagram

	Enrollment	Month												
		1	2	3	4	5	6	7	8	9	10	11	12	>12
Eligibility screening	X													
Informed Consent	X													
Randomization	X													
ASSESSMENTS & OUTCOMES														
Alive and in care at 12 months														X
Number of care visits attended														X
Demographic information	X												X	
Height	X												X	
Weight	X	X	X	X	X	X	X	X	X	X	X	X	X	
WHO Stage	X						X						X	
HIV related diagnoses	X	X	X	X	X	X	X	X	X	X	X	X	X	
HIV knowledge and beliefs	X												X	
HIV-related stigma	X												X	
HIV disclosure	X												X	
Social and family support	X												X	
Depression	X												X	
Alcohol and drug use	X						X						X	
Sexual risk behavior	X						X						X	
Food insecurity	X												X	
ART adherence	X						X						X	
Plasma HIV-1 RNA level	X						X						X	
CD4 T cell count	X						X						X	
Tenofovir-DP level							X						X	
Sexually transmitted infections	X						X						X	
Pregnancy	X						X						X	
Health care utilization and costs	X						X						X	

### Study population: eligibility, recruitment, and enrollment

Study eligibility criteria include: female; 16–23 years of age; not currently on ART or on ART ≤ 3 months; participant knowledge of HIV infection; willing to receive care at the clinic or in the community; and willing to provide consent or assent. Exclusion criteria include: pregnancy at enrollment; a severe HIV/AIDS-related illness requiring hospitalization or intensive medical follow-up; and clinician determination of a developmental stage not suited for study participation. Enrollment was limited to adolescent girls and young women ages 16–23 years because this demographic group represents 80% of AYLWH in Haiti [16–18].

Participants are recruited from those who self-present for HIV testing at GHESKIO, those referred to GHESKIO for HIV testing by a community health worker, and those who receive an HIV test at GHESKIO’s mobile clinic. Potential participants are referred to the GHESKIO Adolescent Clinic, where clinic staff provide post-test counseling, medical assessment, same-day ART initiation, and management of opportunistic infections, per national guidelines, for adolescents and young adults up to 23 years of age [19, 20]. Research staff provide an overview of the study, invite eligible patients to enroll, and assess understanding of the study.

Young adults 18 years and older provide written consent; adolescents 16–17 years old provide written assent with written parental or guardian consent. Following enrollment, participants are randomized to the FANMI model or standard care by computer-generated randomization with block size of 10. Participants may deconsent to participating in the study at any time during the duration of study follow-up.

### HIV services in both study arms

HIV services are provided to participants in both study arms according to World Health Organization (WHO) and Haitian guidelines [19, 20]. Newly diagnosed adolescents receive post-test counseling, are screened for opportunistic infections including TB, and initiated on ART. At monthly visits, participants are monitored for new symptoms, assessed for medication toxicity, and receive one-month medication refills. Prior to October 2018, patients were initiated on first-line ART regimens consisting of tenofovir, lamivudine, and efavirenz. Based on changes to Haiti’s national guidelines, patients were switched to first-line regimens of tenofovir, lamivudine, and dolutegravir starting in November 2018. Second-line ART regimens include tenofovovir and lamivudine with a ritonavir-boosted protease inhibitor, either lopinavir or atazanavir. Plasma HIV-1 viral load is measured at 3, 6 and 12 months after ART initiation; individuals with plasma HIV-1 RNA level > 1000 copies/ml meet with a nurse and social worker for an adherence assessment, development of an individualized adherence plan, and repeat viral load testing 3 months later. Family planning counseling and contraceptive methods are offered to all participants. Girls who become pregnant after enrollment continue to receive HIV services at the Adolescent Clinic or in FANMI groups, in addition to three antenatal care visits at GHESKIO’s Obstetric Clinic.

All participants receive GHESKIO’s routine retention support services, which include transportation fees (~ 100 Haitian gourdes/~$1.40 USD per visit), phone call reminders for scheduled visits, and home visits for missed appointments. When a patient turns 24 years old, Adolescent Clinic staff initiate a transition toGHESKIO’s adult HIV clinic through 3 counseling sessions with a social worker.

**Fig. 2:**
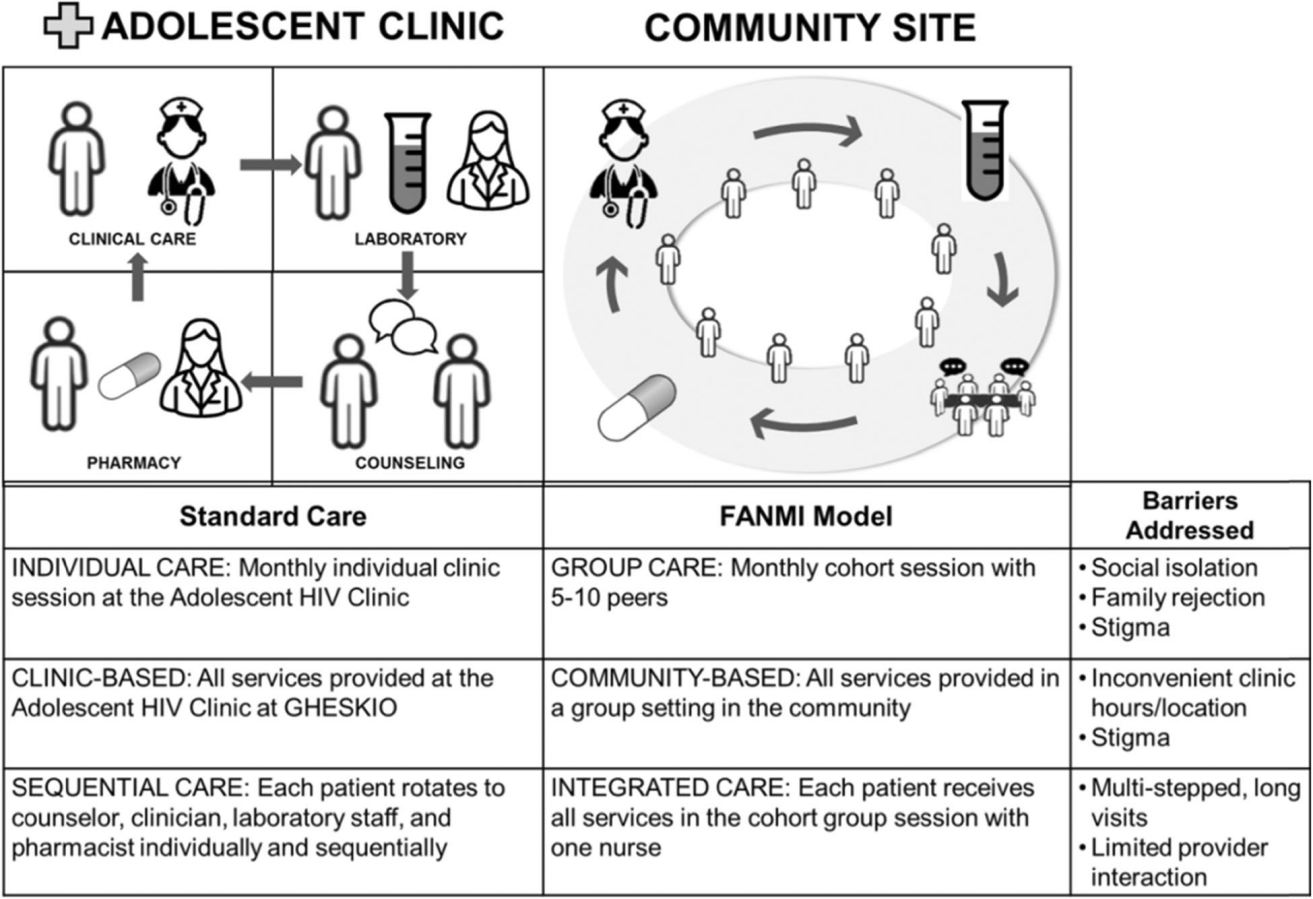
Comparison of the FANMI Model with Standard Care for Adolecent Girls and Young Women Ages 16–23 Living with HIV in Haiti

Figure 2 compares the characteristics of the FANMI model and standard care.

#### Standard care study arm

Participants randomized to standard care receive usual HIV care (as described above) at GHESKIO’s Adolescent Clinic. Study participants see a nurse for clinical care with referral to a physician as needed, and a social worker for individual counseling based on topics decided by the counselor on an ad hoc basis. These steps occur sequentially in separate rooms within the Adolescent Clinic and by different staff members. Typically, each step is separated by waiting time in the Adolescent Clinic waiting room. The participant is then referred to the pharmacy for medication refills and to the laboratory for necessary phlebotomy, both of which are located on the GHESKIO campus but outside GHESKIO’s Adolescent Clinic.

#### FANMI model study arm

Participants randomized to the FANMI model meet with the FANMI nurse who orients them to the community center where they will attend monthly HIV cohort care meetings. Cohort care consists of grouping participants in cohorts that meet monthly for HIV care. The community center space is a free-standing “clubhouse” located on the campus of a local school adjacent to the GHESKIO campus that has a separate entrance from the clinic. Participants are assigned to a FANMI group and informed of their next scheduled group meeting. FANMI groups are formed when there are at least 2 participants available to join. Additional participants are added until the group reaches a total of 5–10 participants or the group has met for 3 months after it was initially formed, whichever occurs first. The participant receives the nurse’s contact information to arrange any additional visits for clinical care and/or counseling as needed. Additional visits outside monthly meetings occur in the FANMI community space unless the participant requires specific clinical care in the Adolescent Clinic.

Each monthly FANMI group visit consists of 30 min of peer socialization followed by 30–45 min of peer-facilitated group counseling led by the FANMI nurse and social worker. During peer socialization, the nurse meets with each participant to review any new symptoms, provide medication refills, and conduct any necessary phlebotomy. During group counseling, the social worker and nurse use a curriculum that includes WHO and GHESKIO counseling messages on topics such as HIV knowledge, retention and ART adherence, stigma, reproductive health and disclosure, tailored to AYLWH (Table 2).

**Table 2:** FANMI HIV Counseling Curriculum Topics

Month^a^	Topics
1	Introduction to FANMI and to HIV
2	Adherence to ART and stigma
3	Problem solving skills and stress management
4	Productive coping skills and resilience
5	Family and social support
6	Reproductive health
7	Sexual risk behavior
8	Disclosure to friends, family and others
9	Gender-based violence
10	Depression and mental health
11	Self-esteem and life goals
12	Celebration of 1 year in HIV care and initiation of transfer to adult clinic

^a^The order of topics covered is modified as needed to fit the needs of each group.

General HIV knowledge and ART adherence materials are covered at every visit

### Study outcomes and measures

The primary outcome is retention in care defined as being alive and retained in care at 12 months with a visit between 11 and 13 months after study enrollment. Secondary outcomes include: viral suppression as defined by plasma HIV-1 RNA viral load <1000 copies/mL at 12 months after enrollment; sexual risk behavior, including self-reported sexual activity, condom use, incidence of sexually-transmitted infections, and pregnancy; acceptability of the FANMI intervention; and health care utilization and costs .

All participants complete a baseline questionnaire at the time of study enrollment, which includes socio-demographic, clinical, and behavioral data. Socio-demographic data includes parent/guardian information, schooling and level of education completed, employment, marital and housing status, and time and cost to travel to GHESKIO. Clinical variables include height, weight, WHO staging, ART regimen, history of pregnancy, sexually transmitted infections (STIs) (e.g. gonorrhea, chlamydia, and syphilis), CD4 count, and viral load. Behavioral measures listed in Table 1 include assessment of HIV testing history, HIV knowledge (HIV Prevention Information Deficits Questionnaire (AHRB)), HIV Stigma Scale for Children (HSSC-8 plus 4 items) [21], Module 75 Disclosure Questionnaire (Special Projects of National Significance Initiative) [22], Multidimensional Scale of Perceived Social Support (MSPSS) [23–25], Center for Epidemiology Studies – Depression Tool (CES-D) [26, 27], Modified Alcohol Use Disorders Identification Test (AUDIT) [27, 28], Short Form Household Food Security Scale [29], Adolescent Sexual Activity Index (ASAI) [30], and a Modified AIDS Clinical Trials Group (ACTG) Adherence Assessment [31].

Six months after enrollment, participants are reassessed for WHO staging, sexual risk behaviors, alcohol and drug use and newly assessed for ART adherence, and health care utilization and costs. Viral load, CD4 count, tenofovir diphosphate levels, pregnancy, and incident gonorrhea and chlamydia are also measured 6 months after enrollment. Twelve months after enrollment, participants are administered the same assessments as were performed at enrollment and 6 months after enrollment, with the addition of tenofovir diphosphate levels (Table 1).

A subset of 30 participants in the FANMI arm will be recruited for qualitative interviews at 6 and 12 months to assess acceptability of the FANMI model. These interviews will be facilitated by a qualitative research assistant not involved in the study. Participants not retained in care at 12 months will be invited to return for a qualitative interview to explore reasons for non-retention and to identify further barriers.

We will also evaluate the fidelity of the intervention by evaluating a subset of 20 randomly selected FANMI group visits for adherence to components of the FANMI model of care. The selected FANMI sessions will be recorded and transcribed and then reviewed using a checklist to evaluate completion and time spent on each component of the intervention including peer socialization, group counseling and clinical care (symptom review, medication adherence assessment, medication refills, and phlebotomy when needed).

### Sample size and power calculations

We based our sample size estimate on a retrospective review of retention from the electronic medical record for the standard care arm and data from a cohort care pilot project for the FANMI arm [32]. We estimate that 85% of participants in the FANMI arm will be alive and retained in care at 12 months, compared to 60% of participants in the standard arm. We calculated the required sample size for this trial using Fisher’s exact test. With a sample size of 160 (80 participants randomized to each arm), we will have ~ 93% power to detect this 25% difference, with an alpha of 5% (for a single primary outcome). Approximately 370 adolescent girls are newly diagnosed with HIV infection at GHESKIO each year. We estimate that 240 of 370 (65%) adolescent girls will satisfy the eligibility criteria and be willing to participate in the study each year. Our sample size also provides us sufficient power to analyze the secondary outcome of achieving viral suppression. With a sample size of 160, we will have > 80% power to detect a 30% difference between the number of participants achieving a viral level < 1000 copies/ml between the two arms. We estimate that 60% of participants in FANMI and 30% of participants in standard care will achieve viral suppression based on pilot data [32].

### Statistical methods

#### Primary outcome

The primary outcome is retention in care at 12 months after enrollment. We anticipate the two study arms will achieve comparability of baseline characteristics and plan to conduct a primary unadjusted analysis. Statistical tests will be two-tailed, with a significance level of 0.05. In the case of imbalance for any baseline variables, we will conduct adjusted analyses and sensitivity analyses using multivariable logistic regression and Cochran-Mantel-Haenszel methods. Results will be reported as proportions and odds ratios, including confidence intervals and statistical significance levels. Categories of non-retention will be described (e.g. death, lost to follow-up), and we will examine differences between the two arms for these categories. We also plan to analyze a cohort effect in the FANMI intervention using generalized estimating equations, where potential within-cohort correlation can be accounted for and intraclass correlation coefficient can be estimated in secondary/sensitivity manner.

#### Secondary outcomes

We will compare the proportion of participants who achieved a viral level < 1000 copies/ml at 12 months between study arms using Fisher’s exact test.

For ‘time-to-event’ outcomes such as pregnancy and STIs, we will use Kaplan-Meier estimates and a Cox proportional hazards model, along with log-rank test and estimation and inference about the hazard ratio. For repeated measures such as condom use, we will use generalized linear mixed effect models that account for correlations within subject. For count data aiming at estimating rate (number of STIs), we will use a Poisson regression model. When data are severely nonlinear, variable transformation (e.g., log) or nonparametric tests with justifications documented will be considered.

All qualitative interviews will be transcribed verbatim, translated in English, and then entered into qualitative software (e.g. Atlas-ti) for coding and analysis. A thematic coding scheme will be created following the main points of the interview guide.

Health care utilization and costs will be summarized as descriptive counts such as number of HIV care visits, laboratory tests, medications, and hospitalizations. Unit costs will be determined for each type of healthcare utilization by applying labor rates and material costs available from GHESKIO and previous studies. Differences in costs between arms will be compared using non-parametric methods if required (e.g. Wilcoxon tests for medians, non-parametric bootstrap for means) due to the skewness often inherent in cost data.

## Discussion

AYLWH are the only age group for which a substantial decrease in AIDS-related mortality has not been achieved [2]. There is an urgent need for differentiated models of HIV care tailored to the needs of high-risk adolescents and youth [33]. The majority of existing differentiated models of adolescent and youth HIV care limit participation to clinically stable patients who have demonstrated adequate ART adherence [14], which by default excludes adolescents who are newly diagnosed or high-risk for poor retention and/or poor medication adherence. We describe a randomized controlled trial evaluating the FANMI model of HIV care, which combines multiple strategies to address the individual and structural barriers to retention in care.

FANMI combines group-based, integrated care in a community setting. It is tailored to young people who are newly diagnosed or not yet defined as stable or adherent. Several other differentiated models of HIV care include group-based care and have shown improvements in retention and virologic suppression among adults and adolescents [34–37] [Willis]. However, these models of care only include patients who are clinically stable having already achieved viral suppression, and are not tailored specifically for the counseling needs of adolescents and young adults [34–36]. Group-based antenatal care has also been successful in improving retention and health outcomes for adolescents in non-HIV settings in the US [38]. FANMI is unique in that it couples group-based care with community-based care among newly diagnosed AYLWH of those who have recently defaulted from care. FANMI builds upon the concept of group-based care by grouping participants in cohorts that meet in the same group each month to foster long-term relationships and social support. As relationships form, providers can adapt the counseling materials to each group’s specific needs to promote sharing and learning between peers, and use group strategies to empower members to manage their health together [39].

Similar to other differentiated care models, FANMI aims to improve health system efficiency by grouping patients together so health workers can treat more patients at once and patients experience shorter waiting times [40–42]. It task-shifts clinical care from physicians to nurses and social workers to redistribute workloads and reduce the number of referrals to physicians. It also leverages the expertise of nurses and social workers in providing in-depth counseling [43]. The FANMI study will add to the limited literature on the feasibility of nurse and social worker-led HIV care for adolescents. Qualitative interviews conducted in the FANMI study will also elucidate provider perspectives on acceptability of this model of care.

By delivering care in a community-setting, FANMI destigmatizes HIV care as participants do not need to attend a clinic known for HIV care, where they may fear being seen by friends or neighbors and suffer from unintended disclosure [44]. It also de-medicalizes HIV care by providing care at a community center, rather than a specialized HIV clinic, and in a group structure where the amount of time spent on counseling compared to time spent on clinical care is increased, and individual clinical consultations are incorporated into group meetings.

Several challenges to recruitment and enrollment have been identified in the early stages of this trial. First, adolescent girls and young women are a hard-to-reach population. Globally, HIV testing is lower in adolescents than in adults, and is particularly low among adolescent girls [45, 46]. In Haiti, only 20% of girls ages 15–19 have ever received an HIV test, compared to 80% of women ages 25–39 [47]. To increase HIV testing and identify eligible participants, we are using three recruitment approaches: 1) widespread community sensitization focused on events and areas where youth are likely to be, 2) mobile testing teams consisting of a nurse, laboratory technician, and two health workers who conduct testing at community sites; and 3) HIV testing among all adolescents who present to GHESKIO clinics for other symptoms such as STIs, tuberculosis, and pregnancy. Second, linking newly diagnosed patients from the community to the clinic for ART initiation and study enrollment is a challenge. We have implemented enhanced post-test counseling, and escort patients directly to the clinic. Similar strategies such as “fast-tracking” patients for ART initiation have been shown to increase linkage to care among adults [48, 49].

Finally, obtaining parental or legal guardian consent for participation of minors in the FANMI study presents obstacles to enrolling patients ages 16–17 years. Many adolescent girls ages 16–17 years refuse disclosing their HIV status to parents or guardians out of fear of rejection, abandonment, violence and loss of housing or economic stability. Other minors live independently, are married or pregnant, or have parents who are unable to travel to the clinic. For girls living with other family members, legal guardianship is rarely formalized, particularly when parents are still alive. Collectively, these barriers exclude an important proportion of minors from research studies that can directly benefit them [50]. The Council for International Organizations of Medical Sciences (CIOMS), WHO, and UNESCO acknowledge the need for waivers of parental consent in circumstances where permission of a parent is not feasible or presents risk to the minor [51]. In countries, such as Haiti, where national laws do not allow for such waivers, UNICEF promotes harmonization of national laws and regulations with international research guidelines in the interests of adolescents who may directly benefit from research [50, 52]. Inclusion of vulnerable minors in research will be critical to improving health outcomes among this key population that is increasingly burdened by HIV.

A limitation of this protocol is that participants on non-nucleoside reverse transcriptase inhibitor (NNRTI) regimen may have accumulated resistance mutations, making viral suppression more difficult to achieve [53] [Walmsley]. Adverse effects of efavirenz such as sleep or neuropsychiatric disturbances may also affect adherence to ART and achievement of viral suppression [54, 55] . As participants are switched to an integrase inhibitor regimen of tenofovir, lamivudine, and dolutegravir, we will measure viral load pre- and post-transition to monitor the impact of dolutegravir on viral suppression.

## Conclusion

Adolescent girls and young women are a vulnerable, underserved population who need tailored models of HIV care that address their unique barriers and challenges to retention in care. The FANMI study aims to improve retention of adolescent girls and young women living with HIV in care by addressing the barriers of stigma, profound social isolation and family rejection, and lengthy clinic visits through a model of community-based cohort care. If shown to be effective, the next step would be a large-scale implementation science study to evaluate scale and sustainability in Haiti and similar resource-poor settings and the potential to tailor this intervention to other high-risk groups.

## Data Availability

This manuscript does not contain any data. Final datasets from this study will be analyzed by investigators at GHESKIO, Weill Cornell Medicine, and University of California Davis. Study findings will be disseminated via publications and communication with local community members.

## Abbreviations

ART: Antiretroviral therapy
AYLWH: Adolescents and youth living with HIV
CAB: Community advisory board
CIOMS: Council for International Organizations of Medical Sciences
GHESKIO: Haitian Group for the Study of Kaposi’s Sarcoma and Opportunistic Infections (French)
STI: Sexually transmitted infection
UNESCO: United Nations Educational, Scientific, and Cultural Organization
WHO: World Health Organization
